# Mechanical diagnosis of human erythrocytes by ultra-high speed manipulation unraveled critical time window for global cytoskeletal remodeling

**DOI:** 10.1038/srep43134

**Published:** 2017-02-24

**Authors:** Hiroaki Ito, Ryo Murakami, Shinya Sakuma, Chia-Hung Dylan Tsai, Thomas Gutsmann, Klaus Brandenburg, Johannes M. B. Pöschl, Fumihito Arai, Makoto Kaneko, Motomu Tanaka

**Affiliations:** 1 Department of Mechanical Engineering, Osaka University, 565-0871 Suita, Japan; 2 Department of Physics, Kyoto University, 606-8502 Kyoto, Japan; 3 Department of Micro-Nano Systems Engineering, Nagoya University, 464-8603 Nagoya, Japan; 4 Research Center Borstel, D23845 Borstel, Germany; 5 Department of Pediatrics, Clinic of Neonatology, University of Heidelberg, D69120 Heidelberg, Germany; 6 Institute of Physical Chemistry, University of Heidelberg, D69120 Heidelberg, Germany; 7 Institute for Integrated Cell-Material Sciences (WPI iCeMS), Kyoto University, 606-8501 Kyoto, Japan

## Abstract

Large deformability of erythrocytes in microvasculature is a prerequisite to realize smooth circulation. We develop a novel tool for the three-step “Catch-Load-Launch” manipulation of a human erythrocyte based on an ultra-high speed position control by a microfluidic “robotic pump”. Quantification of the erythrocyte shape recovery as a function of loading time uncovered the critical time window for the transition between fast and slow recoveries. The comparison with erythrocytes under depletion of adenosine triphosphate revealed that the cytoskeletal remodeling over a whole cell occurs in 3 orders of magnitude longer timescale than the local dissociation-reassociation of a single spectrin node. Finally, we modeled septic conditions by incubating erythrocytes with endotoxin, and found that the exposure to endotoxin results in a significant delay in the characteristic transition time for cytoskeletal remodeling. The high speed manipulation of erythrocytes with a robotic pump technique allows for high throughput mechanical diagnosis of blood-related diseases.

Erythrocytes are known for their significant tolerance against large shape deformation in microvasculature, many of which are much narrower compared to their average diameter. Since ample evidence suggested a tight correlation between the deformability and plasticity in cell deformation and diseases, the viscoelastic parameters of human erythrocytes have been evaluated by various experimental techniques, e.g., micropipette aspiration^1^,^2^,^3^,^4^, optical tweezers^5^,^6^,^7^,^8^,^9^, and flicker spectroscopy^10^,^11^,^12^,^13^. Although these techniques can provide detailed viscoelastic parameters of erythrocytes, they suffer from low throughput due to manual handling of erythrocytes under microscopy. Cone-plate rheometer^14^,^15^ and microfluidic on-chip platforms^16^,^17^,^18^ have also been used frequently as high throughput evaluation techniques, but the output of these techniques is mainly the “deformability”. This coincides with an aspect ratio of the deformed cell, which is a less informative index merely reflecting the shear modulus. Optical stretcher^19^,^20^ and deformability cytometry^21^,^22^ are recent attempts to overcome the trade-off between the throughput and information content^23^,^24^ with aid of the mathematical calculation of small deformations in these systems^25^,^26^. However, the evaluation of small deformation is not directly relevant to quantify the mechanical response of erythrocytes to the large degree of deformation, which they physiologically experience in microvasculatures. Thus, a high throughput diagnostic tool assessing the mechanical response of erythrocytes under large deformation is strongly demanded.

When erythrocytes undergo a large deformation, global cytoskeletal remodeling plays an important role in the shape adaptation. There have been experimental and theoretical studies demonstrating that the remodeling of spectrin cytoskeleton depends on the concentration of adenosine triphosphate (ATP)^20^ and shearing force^27^,^28^. Recent shape fluctuation analyses confirmed the discrepancy in the fluctuation spectrum between the intact and ATP-depleted states^8^,^29^ and the violation of fluctuation-dissipation theorem^9^,^30^. The characteristic time window, about 100 ms, can be attributed to the dissociation-reassociation process of a single node of the triangular spectrin lattice. Although biochemical factors, such as ATP concentration and protein phosphorylation, regulate such a local dynamics and active small fluctuation, the global remodeling in response to the large deformation requires much longer timescale, which has never been assessed in a systematic manner.

In the present work, we developed a novel high throughput assay to quantify the mechanical response of erythrocytes after spatial constriction mimicking microvasculature environments. One of the technical challenges is the precise cell manipulation inside a narrow channel, because the system should be highly sensitive to sustain a delicate balance between pressure and flow velocity. Here, we utilized a “robotic-pump”^31^,^32^ to manipulate an erythrocyte inside a narrow microfluidic path. The erythrocyte was precisely localized in the microchannel by the combination of an ultra-high speed pressure control unit and a real time visual feedback. This enables one to “catch” an erythrocyte in front of the constriction, “load” it inside for a desired time, and quickly “launch” it from the constriction to monitor the shape recovery over time. Using such a “Catch-Load-Launch” manipulation platform, we compared the shape recovery of human erythrocytes in the presence and absence of ATP. The systematic variation of loading time enables us to uncover the characteristic time window for the global spectrin remodeling. Moreover, as a preliminary model of sepsis, we monitored the mechanical response of erythrocytes in the presence of endotoxin (lipopolysaccharide, LPS).

## Results

### “Catch-Load-Launch”manipulation

**Figure 1:**
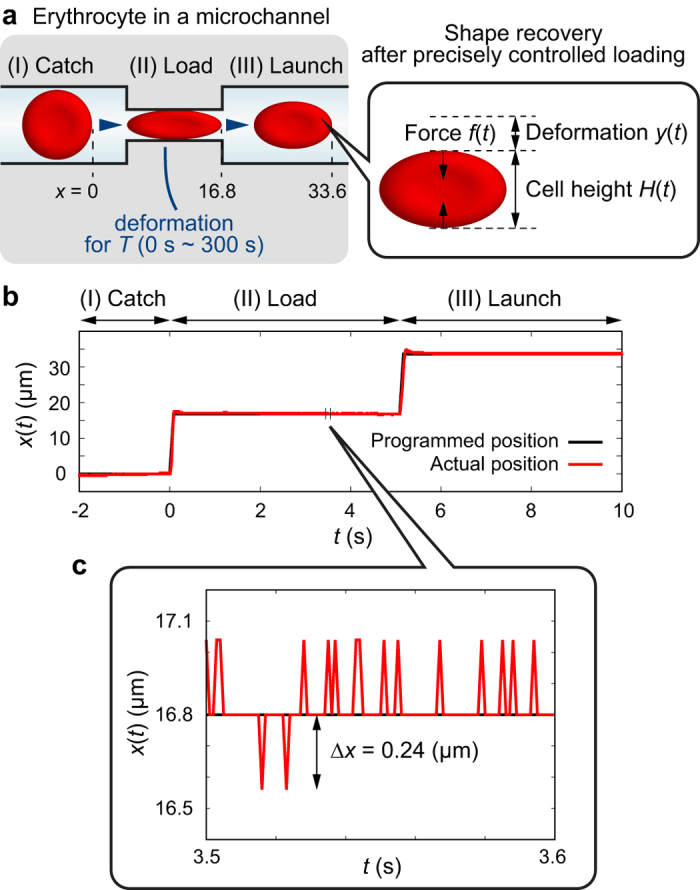
On-chip “Catch-Load-Launch” manipulation of an erythrocyte with the aid of a robotic pump system. (**a**) Schematic diagram of the manipulation. An erythrocyte is first (I) arrested at the entrance (Catch), (II) kept in the narrow path for a distinct time *T* (Load), and (III) released from the narrow path (Launch). (**b**) An example of the actual cell position *x(t*) (red) and the programmed position (black). (**c**) Magnified plot of time window of 100 ms.

Figure 1a schematically represents the “Catch-Load-Launch” manipulation of an erythrocyte on a microfluidic chip. The microfluidic channel with 10 μm × 3.5 μm rectangular cross section has a narrow path in the middle (cross section: 3 μm × 3.5 μm) that mimics the narrowest spatial constriction in microvasculatures of human spleen^33^ to investigate the large deformation of erythrocytes. In the resting state, a biconcave erythrocyte (discocyte) with typical diameter of 8 μm and height of 2 μm is lying in a “thin” microchannel with the height of 3.5 μm. Thus, the projected images of erythrocytes are circular. First, an erythrocyte is caught in front of the narrow path and kept for 2 s to record the original shape (Catch). Second, the cell is transferred into the narrow path and stressed by a constriction force *f(t*) for a programmed time *T* = 0 s, 5 s, 15 s, 30 s, 45 s, 60 s, 120 s, 180 s, and 300 s (Load). Finally, the cell is ejected from the narrow path (Launch). Changes in cell deformation *y(t*) and cell height *H(t*) are recorded as a function of time. The channel length for the launch phase is designed to be 10 μm, which is practically the shortest length for erythrocytes (diameter ≈ 8 μm). It should be noted that the channel is explicitly used for the spatial constriction of the cell position and thus the channel length does not affect the dynamics of shape recovery. Figure 1b exemplifies the cell position *x(t*) recorded in the Catch-Load-Launch manipulation. The actual position (red) precisely follows the programmed position (black) in a step-like way. The programmed velocity d*x*/d*t* in the step-like motion is set to 200 μm/s for sufficiently fast and stable manipulation. The spatial resolution of the manipulation is Δ*x* =  ± 0.24 μm, which corresponds to a minimum pixel size in the present experiment (Fig. 1c).

**Figure 2:**
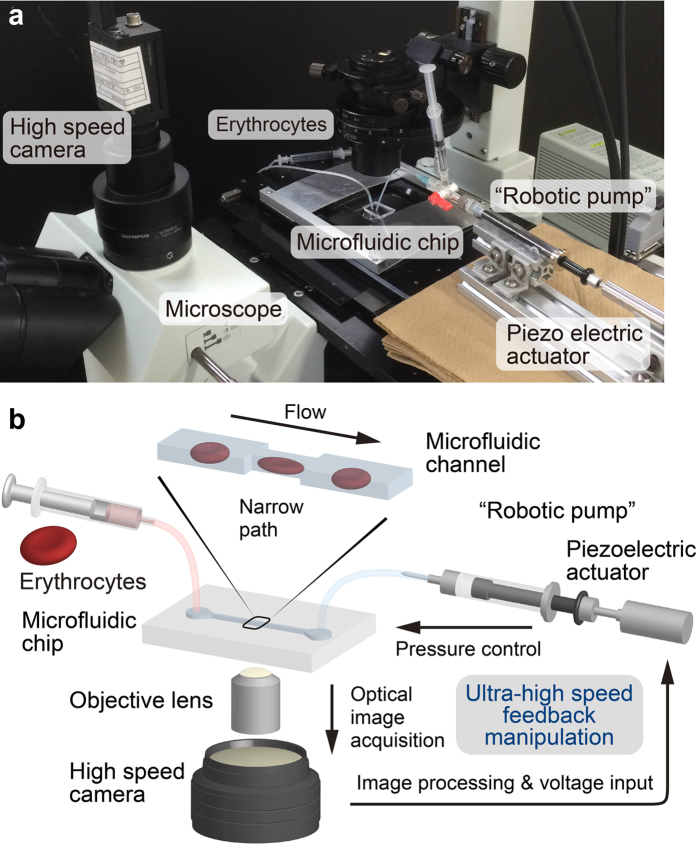
Ultra-high speed feedback system to control the cell position inside a microchannel. (**a**) Experimental setup and (**b**) its detailed schematic diagram. Ultra-high speed (1000 Hz) visual feedback system composed of a high-speed camera and a piezoelectric actuator connected to a syringe provides the pressure control at the channel outlet, enabling the precise cell manipulation (Δ*x* = ± 0.24 μm) inside the narrow path (cross section: 3 μm × 3.5 μm).

Precise, real time control of the cell position *x(t*) is achieved with aid of a “robotic pump” system, enabling the ultra-high speed feedback control of the pressure at the outlet of the microchannel (Fig. 2a, see also Methods for further information). The feedback system is composed of a microscope equipped with a high speed camera and a piezoelectric actuator connected to a syringe. As schematically illustrated in Fig. 2b, the optical images acquired by a high speed camera are processed by a computer, and the detected cell position is converted to voltage information by proportional-integral-derivative (PID) algorithm. The calculated voltage value is then entered into a piezoelectric actuator and further converted to the pressure of a connected syringe, resulting in the manipulation of the cell position *x(t*) within the pixel resolution (Δ*x* =  ±0.24 μm). We set the frequency of image acquisition at 1000 Hz to capture the cell, because the typical residence time of a flowing erythrocyte in the camera view is a few ms. This enables the catch, load, and launch of the cell at an ultra-high spatiotemporal resolution under optical microscopy, which cannot be realized otherwise. The capability not only to manipulate but also to record the image of the target cell every 1 ms is advantageous over other fast spectroscopy^8^,^29^ because one can clearly distinguish the fast elastic response, viscoelastic relaxation, and plastic deformation after the launch.

### Analysis of erythrocyte deformation

**Figure 3:**
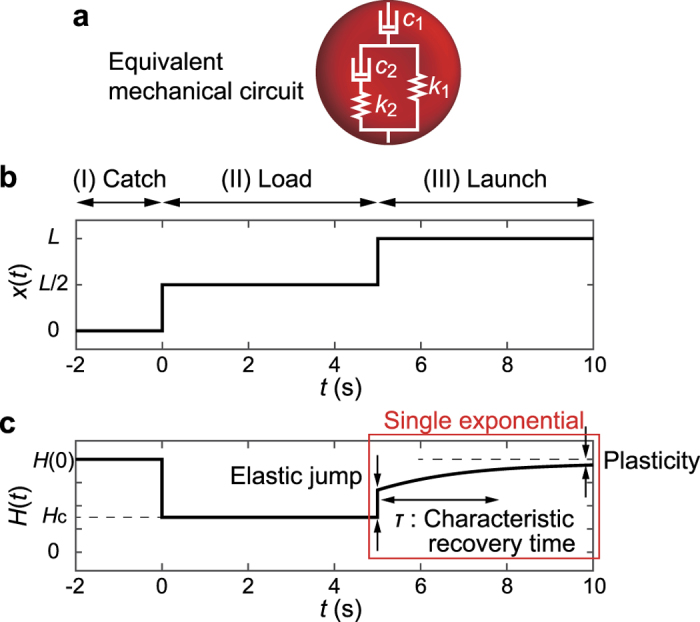
Typical analytical representation of standard linear elastic (SLE) model in (I) Catch, (II) Load, and (III) Launch phases. (**a**) Equivalent mechanical circuit that consists of two springs (spring constants: *k*_1_, *k*_2_) and two dampers (viscous coefficients: *c*_1_, *c*_2_). (**b**) Mathematical representation of the cell position *x(t*) (equation (3)). (c) Changes in cell height *H(t*) (equation (4)), calculated from equations (1) and (3). In the linear viscoelastic regime, the shape recovery in the launching phase (III) (red square in **c**) is represented by a single exponential function. For plotting, parameters are set to *k*_1_ = 1 N/μm, *k*_2_ = 1 N/μm, *c*_1_ = 100 N·s/μm, *c*_2_ = 1 N·s/μm, *H*(0) = 8 μm, and *T* = 5 s despite the arbitrariness of force unit.

After the large deformation inside the narrow path, the launched erythrocyte exhibits a dynamic shape recovery. Although the actual deformation and the shape recovery include both restoration from stretching and folding, we assume a coupled linear viscoelastic elements for mathematical description of the observed cell shape. In fact, the temporal change in cell height obtained from the experimental data could be well explained by a single exponential curve (Fig. 3). Figure 3a shows a typical analytical solution of a standard linear elastic (SLE) model composed of two linear springs (spring constants: *k*_1_ and *k*_2_) and two linear dampers (viscous coefficients: *c*_1_ and *c*_2_). We used the SLE model as the minimal model, since the simpler models such as Voigt model cannot reproduce all the mechanical responses of biological cells, force relaxation, creep, and plastic flow^34^. The constitutive equation of the SLE model is


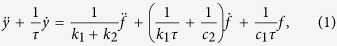


where the characteristic time constant of the shape recovery is represented by


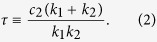


Note that the characteristic recovery time *τ* is independent of the loading time *T*, and therefore an intrinsic material constant. The Catch-Load-Launch manipulation of the cell position *x(t*) can be mathematically approximated as an ideal step function (Fig. 3b, *T* = 5 s as an example):


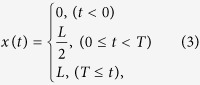


where *L* = 33.6 μm is the total distance that a cell moves from “catch” to “launch”. Figure 3c represents the calculated cell height *H(t*) ( = *H*(0) − *y(t*)) during the manipulation. *H*(0) is the initial height of the target cell measured prior to the loading. *H*_c_ = 3 μm is the constriction width of the narrow path, which corresponds to the cell height in the loading phase. The analytical solution *H(t*) of equation (1) after the launch (*t* ≥ *T*) takes a single exponential function:


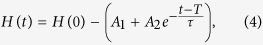


where *A*_1_ and *A*_2_ are constant (see Methods for more details). Thus, *H(t*) is characterized by three information: Elastic jump *H*(0) − (*A*_1_ + *A*_2_) − *H*_c_, plasticity *A*_1_, and characteristic recovery time *τ* (red square in Fig. 3c).

### Shape recovery after launching

**Figure 4:**
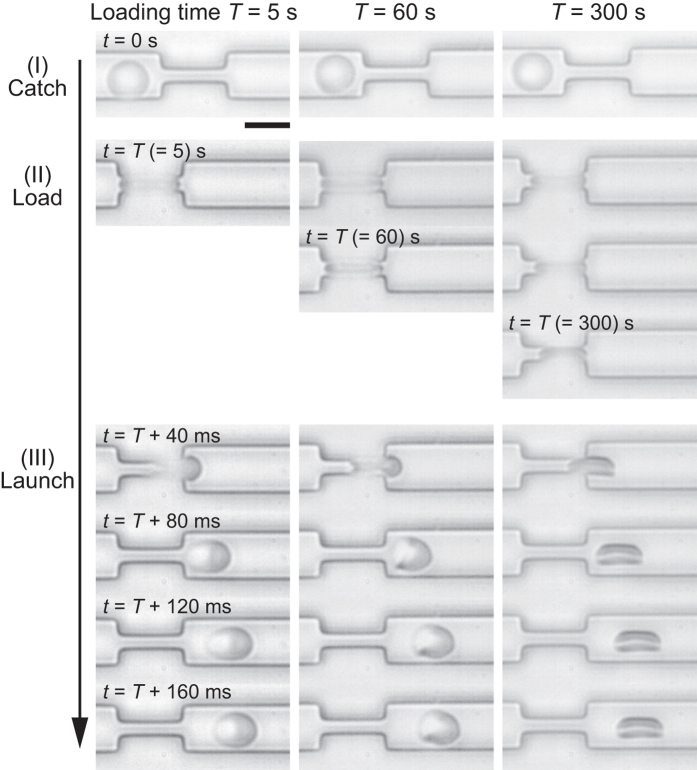
Snapshot images of erythrocytes in “Catch-Load-Launch” process in the cases of loading times *T* = 5 s (left), 60 s (middle), and 300 s (right). After the launch from the narrow path, the cell undergoes shape recovery. The shape recovery becomes drastically slower as the loading time *T* becomes of the order of 100 s. Scale bar is 10 μm.

Figure 4 shows typical snapshot images recorded in the three-step manipulation for various loading times: *T* = 5 s (left), 60 s (middle), and 300 s (right). The data from other conditions (*T* = 0 s, 15 s, and 180 s) are presented in Supplementary Fig. S1. In the catching phase (I), the shape of an erythrocyte projected onto a two-dimensional image is almost circular, corresponding to a normal discocyte. In the loading phase (II), the target cell is largely deformed in a constriction, kept for a specified loading time *T*. The end of the loading at *t* = *T* is the initial condition for shape recovery after the launch. Finally, in the launching phase (III), shape recovery of the ejected cell is monitored as a function of time. When the cell is loaded only for *T* = 5 s, the shape recovery occurs within hundreds of ms. On the other hand, after being kept in the narrow path for *T* = 300 s, the cell no longer recovers its original shape in hundreds of ms. It should be noted that some erythrocytes take triangular or parachute-like shape (e.g., *t* = 60 s + 80 ms in Fig. 4) due to the hydrodynamic or mechanical frictions during the rapid launching^35^,^36^,^37^.

**Figure 5:**
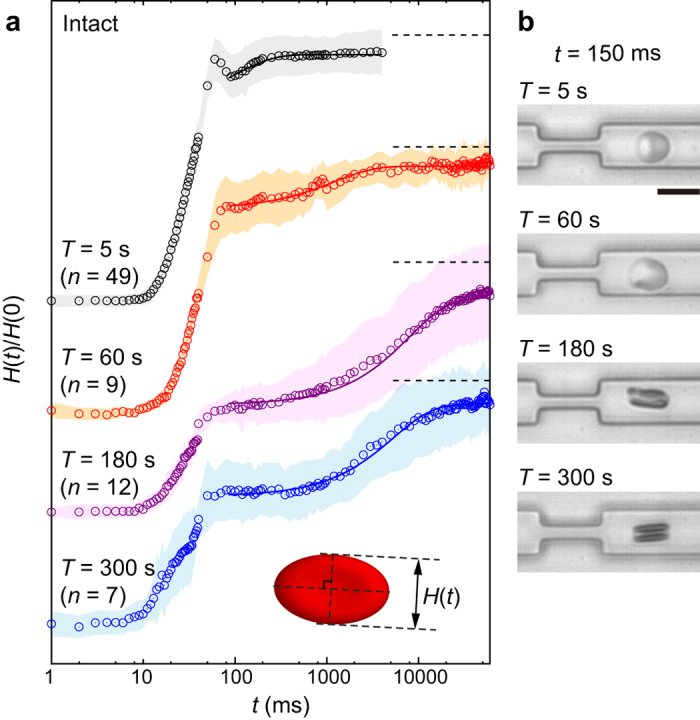
Change in cell height *H(t*) over time, recorded for intact erythrocytes for the different loading times *T*: 5 s, 60 s, 180 s, and 300 s. (**a**) Normalized cell height *H(t*)/*H*(0). The standard deviations and the fitting curves are represented by shaded areas and solid lines, respectively. Dotted lines represent *H(t*)/*H*(0) = 1 for each *T*. Each number of samples *n* is shown above the graph. Inset shows the definition of *H(t*), which is measured along minor axis of the shape. (**b**) Representative snapshot image for each *T*. Scale bar is 10 μm.

Circular symbols in Fig. 5a represent mean temporal changes in the normalized cell height *H(t*)/*H*(0) over 60 s for intact erythrocytes that were loaded in the narrow path for *T* = 5 s, 60 s, 180 s, and 300 s. The data for *T* = 0 s and 15 s are presented in Supplementary Fig. S2. As schematically shown in the inset of Fig. 5a, *H(t*) was determined along the minor axis of the cell at every moment with the aid of real-space imaging. A small but distinct overshoot observed at *t* = *T* + 50–80 ms can be attributed to the geometrical artifact from the cell taking a triangular shape, which is more pronounced for shorter loading time *T* due to significant elastic response. At *t* > 80–100 ms, the cell shape restored front-rear symmetry and exhibited the viscoelastic shape recovery from ellipsoidal to circular, which was subjected to the SLE analysis. Figure 5b shows the representative snapshot images of erythrocytes at *t* = 150 ms after the launch, which corresponds to the various loading time conditions shown in Fig. 5a. We found that the characteristic recovery time increases from *τ*~0.1 s to 10 s in accordance with the increase in the loading time *T* from 5 s to 300 s. A distinct dependence of *τ* on *T* is in clear contrast to the common viscoelastic materials, whose recovery dynamics is independent of the stress duration. It should also be noted that the shape recovery becomes drastically slower (*τ*~10 s) when the loading time *T* becomes longer than the critical loading time *T*_c_~180 s.

### Influence of ATP-depletion

**Figure 6:**
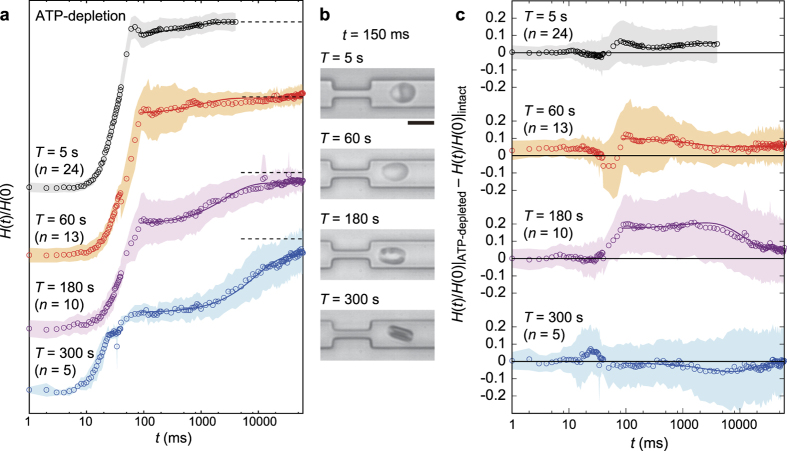
Change in cell height *H(t*) over time, recorded for ATP-depleted erythrocytes for the different loading times *T*: 5 s, 60 s, 180 s, and 300 s. (**a**) Normalized cell height *H(t*)/*H*(0). The standard deviations and the fitting curves are represented by shaded areas and solid lines, respectively. Dotted lines represent *H(t*)/*H*(0) = 1 for each *T*. Each number of samples *n* is shown above the graph. (**b**) Representative snapshot image for each *T*. Scale bar is 10 μm. (**c**) Comparison to intact erythrocytes for each *T*, where *H(t*)/*H*(0) of ATP-depleted erythrocytes exhibited a remarkably faster recovery at *T*~*T*_c_ = 180 s. S.D. (shaded area) is defined as the maximum error coming from each S.D. of ATP-depleted and intact conditions.

The significant deformation and shape recovery through “Catch-Load-Launch” manipulation naturally suggest that such a process is accompanied with the global remodeling of cytoskeletons. As the dynamic remodeling of spectrin cytoskeletons strongly depends on ATP concentrations, we measured the shape recovery of erythrocytes under ATP-depletion (Fig. 6a and b) and compared them with intact erythrocytes (Fig. 6c). Figure 6a shows mean temporal changes in the normalized cell height *H(t*)/*H*(0) over 60 s for ATP-depleted erythrocytes that were loaded in the narrow path for *T* = 5 s, 60 s, 180 s, and 300 s. The data for *T* = 0 s and 15 s are presented in Supplementary Fig. S2. In cases of *T* = 5 s and 60 s, the recovery of the normalized cell height *H(t*)/*H*(0) under ATP-depletion was slightly faster than that of intact erythrocytes, as the difference *H(t*)/*H*(0)|_ATP-depleted_ − *H(t*)/*H*(0)|_intact_ becomes positive (Fig. 6c). This result suggests that the elastic feature becomes relatively significant compared to the viscous feature in ATP-depleted erythrocytes. Moreover, since ATP-depletion impedes the dynamic dissociation and reassociation between spectrin network nodes and cell membranes^38^, our finding suggests that the change in cell mechanics is caused by the more persistent spectrin-membrane bindings due to ATP-depletion. Near the critical time window *T*_c_ = 180 s, ATP-depleted and intact erythrocytes exhibited a remarkable difference in *H(t*), where even the standard deviations coming from the maximum error propagation obviously separate two data sets each other (clear positive difference in Fig. 6c, *T* = 180 s). The significantly different mechanical responses under these biochemical conditions suggest that intact erythrocytes already undergo the global remodeling of spectrin networks in *T*_c_ but ATP-depleted erythrocytes still sustain some spectrin-membrane bindings. Finally, when the cell experiences a longer constriction (*T* = 300 s), the mechanical response of erythrocytes show no clear difference in the recovery time (*τ*~10 s) in the presence and absence of ATP, implying that extensive spatial constriction (*T* = 300 s) causes the global remodeling of cytoskeletons in both cases (Fig. 6c).

**Figure 7:**
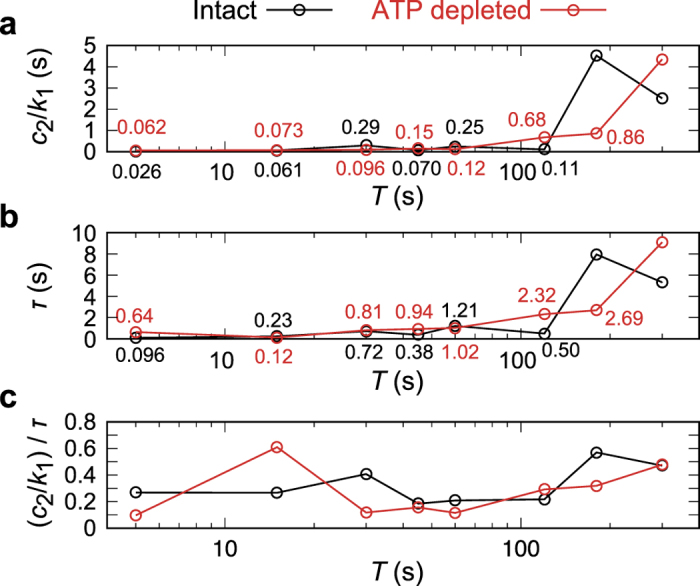
(**a**) *c*_2_/*k*_1_, (**b**) *τ*, and (**c**) (*c*_2_/*k*_1_)/*τ* for intact (black) and ATP-depleted (red) erythrocytes as a function of loading time *T*. According to the increasing loading time, *c*_2_/*k*_1_ and *τ* exhibit a transitional increase at different *T*. The fact that the ratio (*c*_2_/*k*_1_)/*τ* remains almost constant suggests that the global shape recovery is dominated by the internal viscous coefficient *c*_2_.

To quantitatively assess our experimental observations, we evaluated the mechanical parameters and time constants according to equation (4). Solid lines in Figs 5a and 6a correspond to the least squared fittings of the experimental data, showing an excellent agreement with the coefficient of determination *R*^2^ > 0.92 with an only exception *R*^2^ = 0.77 for *T* = 60 s, ATP-depleted erythrocytes. Figure 7 shows the calculated mechanical parameter and time constant as a function of loading times *T*. Note that the data corresponding to *T* = 0 s (Supplementary Fig. S2) are not included as the response was dominated solely by the elastic jump. Here, the spring constant *k*_1_ was fixed to unity as a reference force unit throughout the fittings, because the force exerted to the cell *f(t*) by constriction cannot be measured experimentally. The relative significance of each parameter was systematically checked: *k*_1_ and *k*_2_ stay in the same order of magnitude (Supplementary Fig. S3), while *c*_1_ is more than 3 orders of magnitude larger than *c*_2_. This suggests that both spring constants contribute to determine the elastic properties whereas the viscous property is governed by *c*_2_. The global plasticity of erythrocytes arising from *c*_1_ is actually quite low, as *H(t*)/*H*(0) reaches the saturation level > 0.9 for all the experimental conditions after *t* > 10 s. Thus, we selected *c*_2_/*k*_1_ (Fig. 7a) and *τ* = *c*_2_ (*k*_1_ + *k*_2_)/(*k*_1_*k*_2_) = (*c*_2_/*k*_1_)(1 + *k*_2_/*k*_1_)/(*k*_2_/*k*_1_) (Fig. 7b) as the representative parameters. The fact that the ratio between *c*_2_/*k*_1_ and *τ* remained comparable implies that *c*_2_ determines the global shape relaxation kinetics of erythrocytes (Fig. 7c). As presented in Fig. 7a, *c*_2_/*k*_1_ of intact erythrocytes (black) stays low (*c*_2_/*k*_1_ < 1) until *T* = 120 s. When the loading time *T* exceeds *T*_c_ = 180 s, *c*_2_/*k*_1_ increases to > 1 and thus the shape recovery becomes slower. In case of ATP-depleted erythrocytes (red), the cells did not exhibit remarkable increase in *c*_2_/*k*_1_ < 1 until *T* ≥ 300 s. Such a delay in the transition under ATP-depletion was also found for the characteristic recovery time *τ* (Fig. 7b). In summary, we found that shape recovery occurs rather fast (*τ*_fast_ = 0.1–3 s) at *T* ≤ 120 s. After the longer loading time (*T* = 180 s), recovery time *τ* of intact erythrocytes increases up to *τ*_slow_ ≈ 8 s, while *τ* for ATP-depleted erythrocytes remains lower (*τ* < 3 s). In contrast to the clear changes in *c*_2_/*k*_1_ and *τ*, neither *k*_2_/*k*_1_ or *c*_1_/*k*_1_ exhibits any systematic dependence on *T*, irrespective of the presence and absence of ATP (Supplementary Fig. S3).

### Impact of exposure to endotoxin: sepsis model

**Figure 8:**
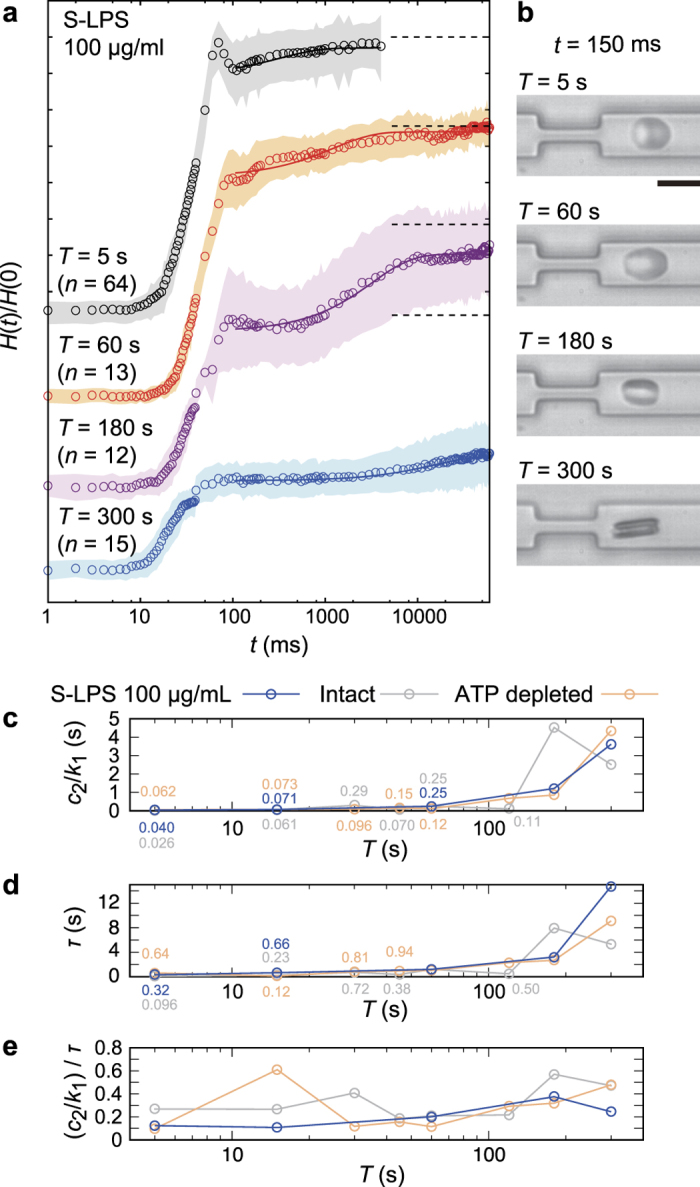
Impact of endotoxin (100 μg/ml S-LPS) on the shape recovery kinetics. (**a**) Normalized cell heights *H(t*)/*H*(0) for the different loading times *T* = 5 s, 60 s, 180 s, and 300 s over time after the exposure to 100 μg/ml S-LPS. The standard deviations and the fitting curves are represented by shaded areas and solid lines, respectively. Dotted lines represent *H(t*)/*H*(0) = 1 for each *T*. Each number of samples *n* is shown above the graph. (**b**) Representative snapshot image for each *T*. Scale bar is 10 μm. (**c**) *c*_2_/*k*_1_, (**d**) *τ*, and (**e**) (*c*_2_/*k*_1_)/*τ* for erythrocytes exposed to 100 μg/ml endotoxin (blue) as a function of *T*. Results from intact (grey) and ATP-depleted (orange) erythrocytes are plotted for comparison.

Finally, we applied our “Catch-Load-Launch” manipulation platform for the potential diagnosis of a blood-related disease: septic shock. Sepsis is a life-threatening disease with systematic inflammatory responses induced by endotoxin (lipopolysaccharide, LPS) molecules released from the outer membranes of Gram-negative bacteria^39^. Although many reports suggested that the disturbance of smooth microcirculation in sepsis is correlated to the “stiffening” of erythrocytes by LPSs^40^,^41^, the mechanical diagnosis has been impeded due to the lack of proper design of high throughput methodologies. Here, we examined if our strategy can detect the impact of lipopolysaccharide from *Sarmonella minnesota* (S-LPS, see also Supplementary Fig. S4 for molecular detail) on the dynamic mechanical response of human erythrocytes. Figure 8a displays *H(t*)/*H*(0) plotted versus *t* (symbols for the mean values, and shaded areas for the standard deviations) and the corresponding fits (solid lines) for *T* = 5, 60, 180, and 300 s, measured in the presence of 100 μg/ml of S-LPS. The data from other loading times are presented in Supplementary Fig. S5. Figure 8b shows the representative snapshot images at *t* = 150 ms after the launch for each loading time *T*. Figure 8c–e (blue) shows the representative parameters (*c*_2_/*k*_1_, *τ*, and their ratio) plotted versus *T*, compared to the results from intact (grey) and ATP-depleted (orange) erythrocytes. The mechanical parameters of S-LPS-influenced erythrocytes are very close to those of ATP-depleted erythrocytes, exhibiting similar shift of the critical loading time *T*_c_. On the other hand, *k*_2_/*k*_1_ and *c*_1_/*k*_1_ exhibited no remarkable tendency for all experimental conditions (Supplementary Fig. S6). It should be noted that exposure to S-LPS for only 15 min is sufficient to cause the change in mechanical response, which is comparable to that of erythrocytes treated for 5 h under ATP-depletion (see Methods), suggesting the significant increase in the cytoskeleton-membrane coupling. This finding is consistent with our previous fluctuation analysis, indicating that LPSs decrease the bending stiffness *κ* and increase the membrane-cytoskeleton coupling constant *γ*^40^. As *γ* is proportional to the shear modulus dominated by the underlying specrin network, the increase in cytoskeleton-membrane coupling observed from both passive fluctuation analysis^40^ and active constriction seems to agree well with the previous cone-plate rheometer experiments, suggesting that LPSs make erythrocytes much less deformable.

## Discussion

Previously, the fast characteristic recovery time *τ*_fast_ in the order of 0.1 s was evaluated from the viscoelastic shape recovery after a local deformation by micropipette aspiration^5^,^42^,^43^, the parachute-shaped deformation by multiple optical trapping^44^, and the entire shear deformation by Couette flow^45^. Recent shape fluctuation analyses suggested that the time window of dissociation-reassociation of a spectrin network is about 100 ms on a single node level^8^,^29^, whose ATP-dependent, non-equilibrium response could be evidenced by the violation of fluctuation-dissipation theorem^9^,^30^. However, although the timescale of fast recovery *τ*_fast_ = 0.1–1 s apparently seems to agree, it is not valid to directly correlate the dissociation-reassociation of a single node and the global shape change. Rather, it seems more plausible to assign *τ*_fast_ as an intrinsic material constant of erythrocytes, reflecting the surface viscosity and shear modulus^42^. On the other hand, an extremely slow shape recovery with a much longer characteristic time window *τ*_ex-slow_~1000 s was also observed after the extensive diametrical aspiration of a cell for 10–100 min^3^. Such an extremely slow process was also found in segregated distribution of lipids, cytoskeleton, and mobile transmembrane proteins under micropipette suction (*τ* > 30 min^46^), existence of “memory” of lateral membrane compositions after applied shear force (*τ* > 4 h^47^), and spontaneous shape transition between discocytes and echinocytes (*τ* > 10 h^48^), but there have been no systematic investigations on the mechanism of the extremely slow phenomena.

**Figure 9:**
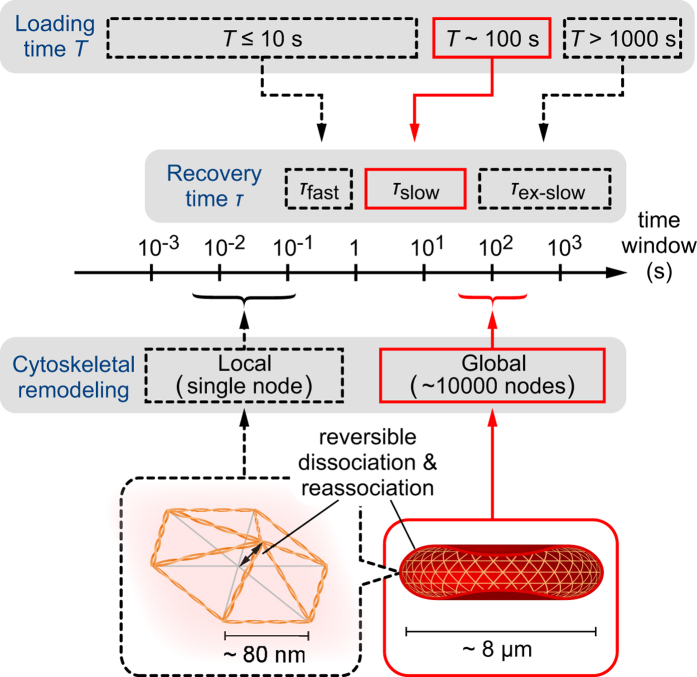
Schematic diagram of characteristic time windows for loading, recovery, and cytoskeletal remodeling. Global cytoskeletal remodeling in 100 s and the corresponding loading time *T*_c_ result in the slow shape recovery with the characteristic time *τ*_slow_~10 s, as revealed by the present study (denoted by red). The global remodeling of entire spectrin network needs much longer time by 3 orders of magnitude compared to the local remodeling by ATP-dependent reversible dissociation-reassociation of a single node of the triangular lattice.

The experimental strategy proposed in the present work enables to fill the gap between the fast (*τ*_fast_ = 0.1–1 s) and extremely slow (*τ*_ex-slow_~1000 s) shape recovery, unraveling a new time window reflecting the “global” cytoskeletal remodeling. An increasing number of studies suggested that the ATP-dependent deformation of a erythrocyte is originated from the cytoskeletal remodeling by using non-equilibrium contour fluctuation analysis^8^,^9^,^13^,^29^,^30^,^38^,^40^ and shear deformation experiments^27^,^28^,^47^. Therefore, the significant delay of *T*_c_ found for ATP-depleted erythrocytes can be attributed to the decrease in the connectivity between spectrin networks and the associated proteins, such as protein 4.1R^49^, band-3^50^,^51^, ankyrin^52^, actin^53^, and spectrin itself^54^. Although exact molecular details are still not clearly identified, our results imply that the slow relaxation (relaxation time: *τ*_slow_~10 s) accompanied by the loading for *T*_c_ ≈ 180 s coincides with the ATP-dependent global remodeling of spectrin cytoskeletons (Fig. 9). This time window is 3 orders of magnitude longer compared to both the fast response (*τ*_fast_~0.1 s) and the local dissociation-reassociation of a single cytoskeletal node (~0.1 s), dissecting the global and local dynamics. It should be noted that the measurements of ATP-dependent fluctuation are based on small displacements of the cell rim from its average discocyte shape, which is completely different from the constriction-induced, whole deformation designed in this study that mimics the physiological cell deformation during microcirculation.

In this study, we combined microfluidics and a “robotic pump” system for the mechanical diagnosis of human erythrocytes. By monitoring the dynamic recovery of cell height *H(t*) upon launching from the narrow path with the aid of a high-speed camera and a visual feedback pressure controller, we could dissect a characteristic loading time *T*_c_ for the global cytoskeleton remodeling in the absence and presence of the source of chemical energy (ATP) and endotoxin (S-LPS), which significantly influence the erythrocyte viscoelasticity. Ample evidence suggested a tight correlation between the viscoelastic cell deformation and diseases. For example, sepsis, a systematic inflammatory response syndrome, is caused by the exposure to endotoxin (LPSs), leading to a significant decrease in the deformability. Such a stiffening, characterized by an increase in the shear modulus and thus disturbance of microcirculatory, is much more pronounced for erythrocytes of neonates than those of adults, which makes sepsis more critical for neonates with pre-matured immune systems^40^,^41^,^55^. Another prominent example is the increase in shear modulus of erythrocytes by the progression of malaria infection, demonstrated by micropipette aspiration^56^, optical tweezers^57^, and hydrodynamic shear^58^. As a preliminary attempt to diagnose erythrocytes in a diseased state, we monitored the mechanical response of erythrocytes in the presence of endotoxin S-LPS, mimicking the septic shock. The exposure to 100 μg/ml toxin for 15 min caused a distinct shift in *T*_c_ to the longer loading time, which is the same tendency as the mechanical response under ATP-depletion. This finding can be attributed to the inhibited plasticity in membrane-cytoskeleton coupling. Therefore, we concluded that our “Catch-Load-Launch” manipulation platform has a large potential towards the mechanical diagnosis of diseased blood cells.

One of the major problems in mechanical cell diagnosis is the limitation in throughputs. Among several techniques developed in the past years, optical stretcher partly overcame the problem as the technique is non-invasive and can be coupled to a flow chamber^19^,^20^. However, optical stretcher has a size limitation of the object diameter (6–30 μm) originating from laser-focus size. Moreover, the applied force cannot be constant during the trapping of a deformable object as the momentum transfer from a laser varies according to the deformation of the object surface. In contrast, our “Catch-Load-Launch” manipulation system is applicable for any object sizes greater than the resolution of microfabrication (~1 μm). Moreover, our system specifies the constant deviation amplitude but not the force amplitude, which is physiologically more relevant to the constriction inside microvasculature. The exact deviation control at ultra-high frequency (1000 Hz) and spatial precision (Δ*x* = ± 0.24 μm) under the pressure control by the robotic pump enables the actual step-like deformation (Fig. 1b), and thus we can access a wide response time window (1 ms–100 s) in real-space imaging. By utilizing these unique advantages we found that the recovery time *τ* increases from *τ*_fast_~0.1 s to *τ*_slow_~10 s according to the increase in the loading time *T* from *T* = 0 s to *T* = 300 s through the critical timescale *T*_c_ = 180 s, which is the characteristic time window for the global remodeling of cytoskeleton. Although the present experiment does not provide the absolute values of these parameters due to the lack of force measurements, the implementation of a force sensor and/or movable wall to apply a specified force in the on-chip robotic pump system could further improve the platform in terms of sensitivity and information content. Last but not least, it should be pointed out that the throughput in our first demonstration (several tens of cells per hour in average) was slightly lower than fully automated optical stretcher equipment (50–100 cells per hour^59^). However, further optimization of the large degree of freedoms of experimental conditions such as cell density, flow speed, programmed procedure, etc., will open up a novel way for the mechanical diagnosis of blood-related diseases based on dynamic response of erythrocytes to spatial constriction.

## Methods

### Sample preparation

Adult blood was drawn from healthy donors based on the informed consent. All the experiments and experimental protocols for erythrocyte-deformability tests in microchannels were approved by the Ethical Committee of Osaka University and performed following the guideline and regulation. Immediately after the blood drawing, the blood was dispersed at a concentration of 1% (v/v) in standard saline for intact and ATP-depleted conditions, and at the same concentration in saline containing 100 μg/ml wild-type lipopolysaccharide (S-LPS) for S-LPS influenced condition. ATP-depletion was performed by incubating the above-described 1% blood solution at 37 °C for 5 h. The ATP concentration of the ATP-depleted erythrocyte measured by luciferin-luciferase assay was 43.8 ± 4.4% of an intact erythrocyte.

### Fabrication of microfluidic chips

Microfluidic channel was fabricated in a microfluidic chips made of poly(dimethylsiloxane) (PDMS) resin (SILPOT 184, DOW CORNING TORAY) bounded to a glass substrate. The width of the microchannel, narrow path, and the height of the channel structure were designed as 10 μm, 3 μm, and 3.5 μm, respectively. A master mold for replicating PDMS chips was made by negative photoresist SU8–3005 via standard laser photolithography. PDMS and the curing agent were mixed at a ratio 9:1, and the mixture was degassed under vacuum for 30 min before being poured onto the mold. After the degasification of the PDMS mixture with the mold for another 10 min, the PDMS was baked at 90 °C for 1 h. Afterwards the mold was removed, and two holes for inlet and outlet were punched on the PDMS chip. PDMS surface was treated by plasma cleaner (CUTE 1MP, Femto Science) and bonded on a glass substrate. The inlet and outlet of the fabricated chip were connected to a syringe filled with the above described diluted erythrocyte solution and “robotic pump” (see Fig. 2 and next paragraph) filled with the standard saline, respectively, via Ethylene tetrafluoroethylene (ETFE) tubes and silicone tubes. Each erythrocyte was deformed and evaluated inside the channel one by one.

### Pressure control and observation

Flow of the solution inside the microchannel is basically driven by a constant pressure difference between the inlet and outlet of the channel simply maintained by the atmosphere pressure and gravitational force. The additional pressure control for the temporal cell manipulation was performed by an outlet syringe automatically driven by a connected piezoelectric actuator (PSt 150/5/40 VS10, PIEZOMECHANIK) controlled by a proportional-integral-derivative (PID) algorithm coded in C for processing camera acquisition, cell position detection, voltage input to the piezoelectric actuator through a voltage amplifier (M-2655, MESS TEK), and recording the monitored images, called “robotic pump” in this paper. To capture the exact time window of shape recovery widely ranging from fast elastic response (of the order of ms) and slow viscoelastic relaxation (of the order of min), the input of image was taken at every 1 ms and the output was set at every 1 ms for 0 ms ≤ *t* < 40 ms, 10 ms for 40 ms ≤ *t* < 200 ms, 50 ms for 200 ms ≤ *t* < 1000 ms, 200 ms for 1000 ms ≤ *t* < 2000 ms, 500 ms for 2000 ms ≤ *t* < 4000 ms, and 1000 ms for 4000 ms ≤ *t* ≤ 60000 ms after launch. In the cases of shorter loading times *T* = 0 s, 5 s, and 15 s the images were recorded up to 5000 ms, while in the cases of longer loading times *T* = 30 s, 45 s, 60 s, 120 s, 180 s, and 300 s, the images were recorded up to 60000 ms.

For the feedback control and observation, the microfluidic chip was mounted on a microscope (IX71, Olympus) with a × 40 objective lens (N.A. = 0.6) connected to a high-speed camera (IDP-Express R2000, Photron). The resultant pixel size of image acquisition was 0.24 μm, which is the spatial precision itself of the manipulation. The camera view was set around the narrow path of the microchannel, and the recording frequency was set at 1000 Hz, which makes it possible to manipulate the cell position in the rapid flow in the thin microchannel by the visual feedback pressure controller. Using this system we performed the three-step Catch-Load-Launch manipulation (see main text).

### Data analysis

Obtained sequential images were processed in a custom routine developed in MATLAB R2015a with Image processing toolbox (MathWorks). Using the image in the catching phase, data screening was conducted for the cell with high ellipsoidal eccentricity ( > 0.5) to avoid the analyses of unexpected asymmetric deformation inside the narrow path. Then the cell shape and height in the launching phase was measured based on the binarized images calculated from the original bright field images. The changes in cell orientation after launch was corrected in every image before the height calculation based on the angular degree of the ellipsoidal minor axis of the binarized cell. Least square fittings of the cell height changes for various loading time *T* = 0 s, 5 s, 15 s, 30 s, 45 s, 60 s, 120 s, 180 s, and 300 s with equation (4) was performed using the mean cell height data after 80 ms (standard saline and that with ATP-depletion assay) and for *T* = 0 s, 5 s, 15 s, 60 s, 180 s, and 300 s after 100 ms (standard saline dissolving 100 μg/ml S-LPS) when the cells stopped initial elastic responses and entered the viscoelastic regime in the launching phase.

### More details of SLE model

We derived analytical solution of standard linear elastic (SLE) model in the launching phase in Catch-Load-Launch experiment. The deformation length *y(t*) becomes a single exponential function with a constant offset *A*_1_ and a constant coefficient *A*_2_:


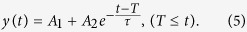


With the mechanical parameters *k*_1_, *k*_2_, *c*_1_, and *c*_2_, these constants *A*_1_ and *A*_2_ are expressed as


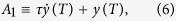



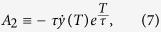


where


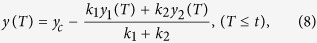



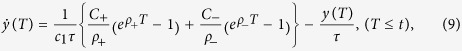



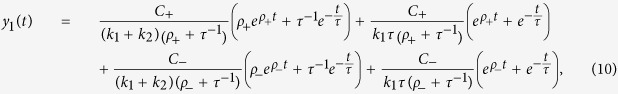



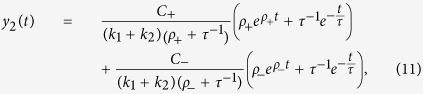



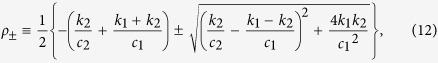



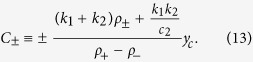


Here *y*_c_ is the deformation length by the constraint in the narrow path of the channel with *H*_c_ = 3 μm in width, namely, *y*_c_ = *H*(0) − *H*_c_. In the analyses in this study, we fixed a parameter *k*_1_ as unity for each loading time condition, and evaluated the ratios between other parameters *k*_2_/*k*_1_, *c*_1_/*k*_1_, and *c*_2_/*k*_1_. The validation of this assumption is discussed with the results in main text.

## Additional Information

**How to cite this article**: Ito, H. *et al*. Mechanical diagnosis of human erythrocytes by ultra-high speed manipulation unraveled critical time window for global cytoskeletal remodeling. *Sci. Rep.* **7**, 43134; doi: 10.1038/srep43134 (2017).

**Publisher's note:** Springer Nature remains neutral with regard to jurisdictional claims in published maps and institutional affiliations.

## Supplementary Material

Supplementary Information
