# Supplementary material for: Mechanical diagnosis of human erythrocytes by ultra-high speed manipulation unraveled critical time window for global cytoskeletal remodeling
Source: Sci Rep. 2017 Feb 24;7:43134. doi: 10.1038/srep43134 (PMC5324053; doi:10.1038/srep43134)
Supplement: Supplementary Information [file srep43134-s1.doc]

**Supplementary Information**

**Mechanical diagnosis of human erythrocytes by ultra-high speed manipulation unraveled critical time window for global cytoskeletal remodeling**

Hiroaki Ito,^1,2^ Ryo Murakami,^1^ Shinya Sakuma,^3^ Chia-Hung Dylan Tsai,^1^ Thomas Gutsmann,^4^ Klaus Brandenburg,^4^ Johannes M. B. Pöschl,^5^ Fumihito Arai,^3^ Makoto Kaneko^1,*^ & Motomu Tanaka^6,7,*^

^1^Department of Mechanical Engineering, Osaka University, 565-0871 Suita, Japan

^2^Department of Physics, Kyoto University, 606-8502 Kyoto, Japan

^3^Department of Micro-Nano Systems Engineering, Nagoya University, 464-8603 Nagoya, Japan

^4^Research Center Borstel, D23845 Borstel, Germany

^5^Department of Pediatrics, Clinic of Neonatology, University of Heidelberg, D69120 Heidelberg, Germany

^6^Institute of Physical Chemistry, University of Heidelberg, D69120 Heidelberg, Germany

^7^Institute for Cell-Material Sciences (WPI iCeMS), Kyoto University, 606-8501 Kyoto, Japan

*Correspondence and requests for materials should be addressed to M. K. (mk@mech.eng.osaka-u.ac.jp) and M. T. (tanaka@uni-heidelberg.de)


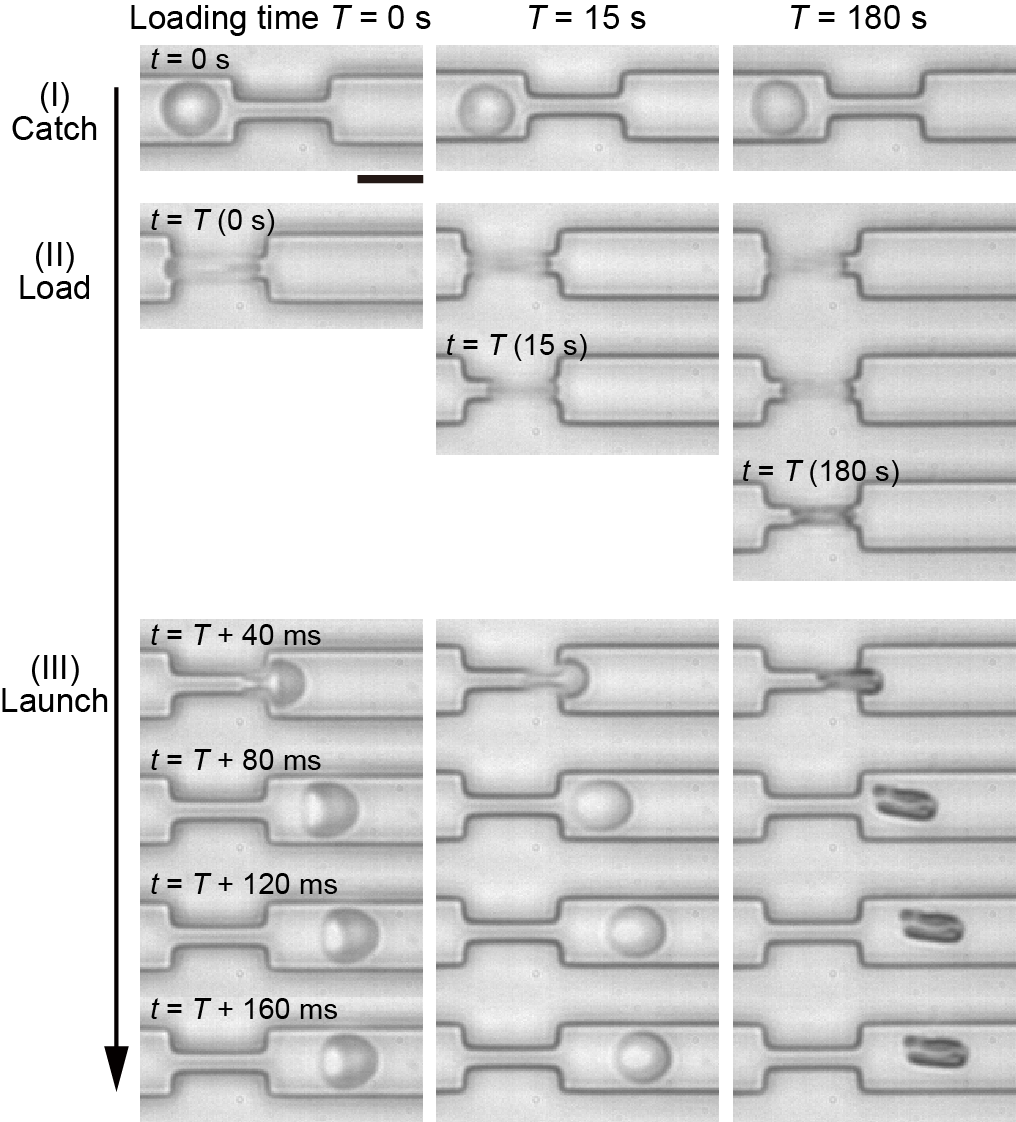


**Figure S1 | Snapshot images of erythrocytes in “Catch-Load-Launch” process in the cases of loading times *T* = 0 s, 15 s, and 180 s.** The shape recovery becomes drastically slower as the loading time *T* becomes longer than the critical loading time *T*_c_ ~ 180 s. See also Fig. 4 in the main text. Scale bar in the left top image is 10 μm.


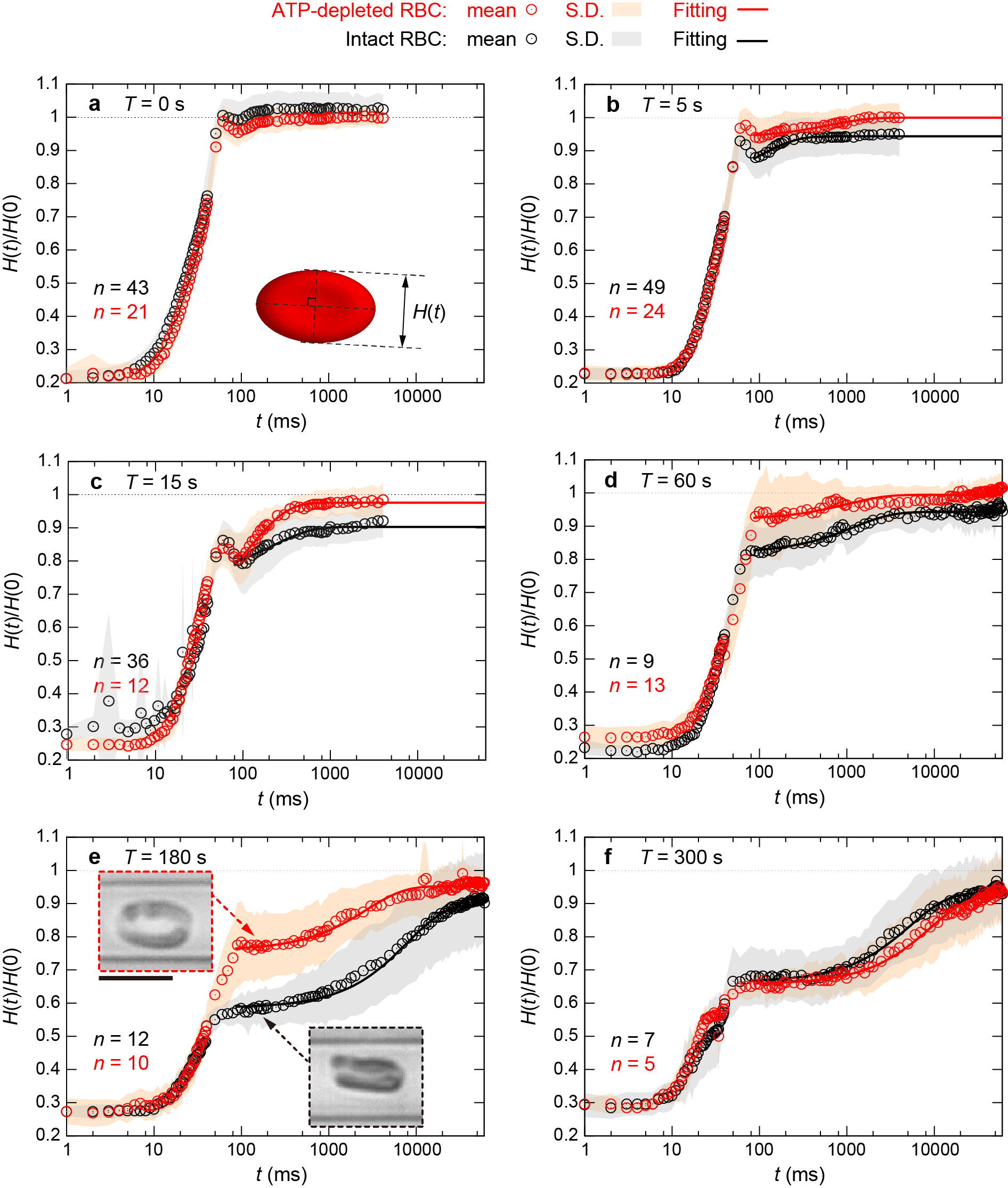


**Figure S2 | Normalized intact (black) and ATP-depleted (red) cell heights for the loading times (a) *T* = 0 s, (b) 5 s, (c) 15 s, (d) 60 s, (e) 180 s, and (f) 300 s as a function of recorded time *t* in (III) launching phase.** Mean values of the measured cell heights *H*(*t*)/*H*(0), the standard deviations, and the fittings with a theoretical function are represented by circular symbols, shades, and solid lines, respectively for each loading time. Each number of samples *n* is given above the graph.


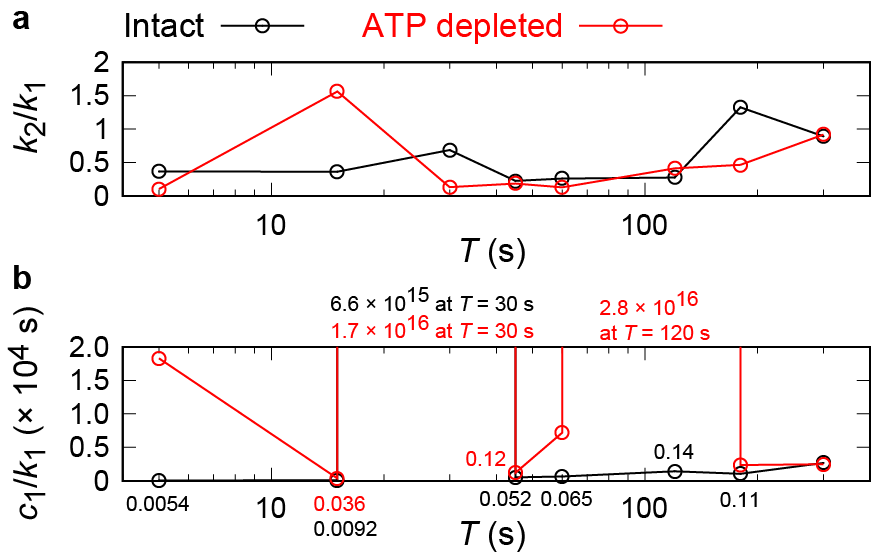


**Figure S3 | Estimated (a) *k*_2_/*k*_1_ and (b) *c*_1_/*k*_1_ for intact (black) and ATP depleted (red) erythrocytes as a function of a widely ranged loading time *T* from seconds to hundreds of seconds.** Divergence of *c*_1_/*k*_1_ means that erythrocytes exhibited no plasticity. *k*_2_/*k*_1_ and *c*_1_/*k*_1_ do not exhibit any clear systematic tendency among different loading time *T* and different metabolic conditions, contrary to *c*_2_/*k*_1_ and *τ* accompanied by the drastic transition by 2 orders of magnitude (see Fig.6).


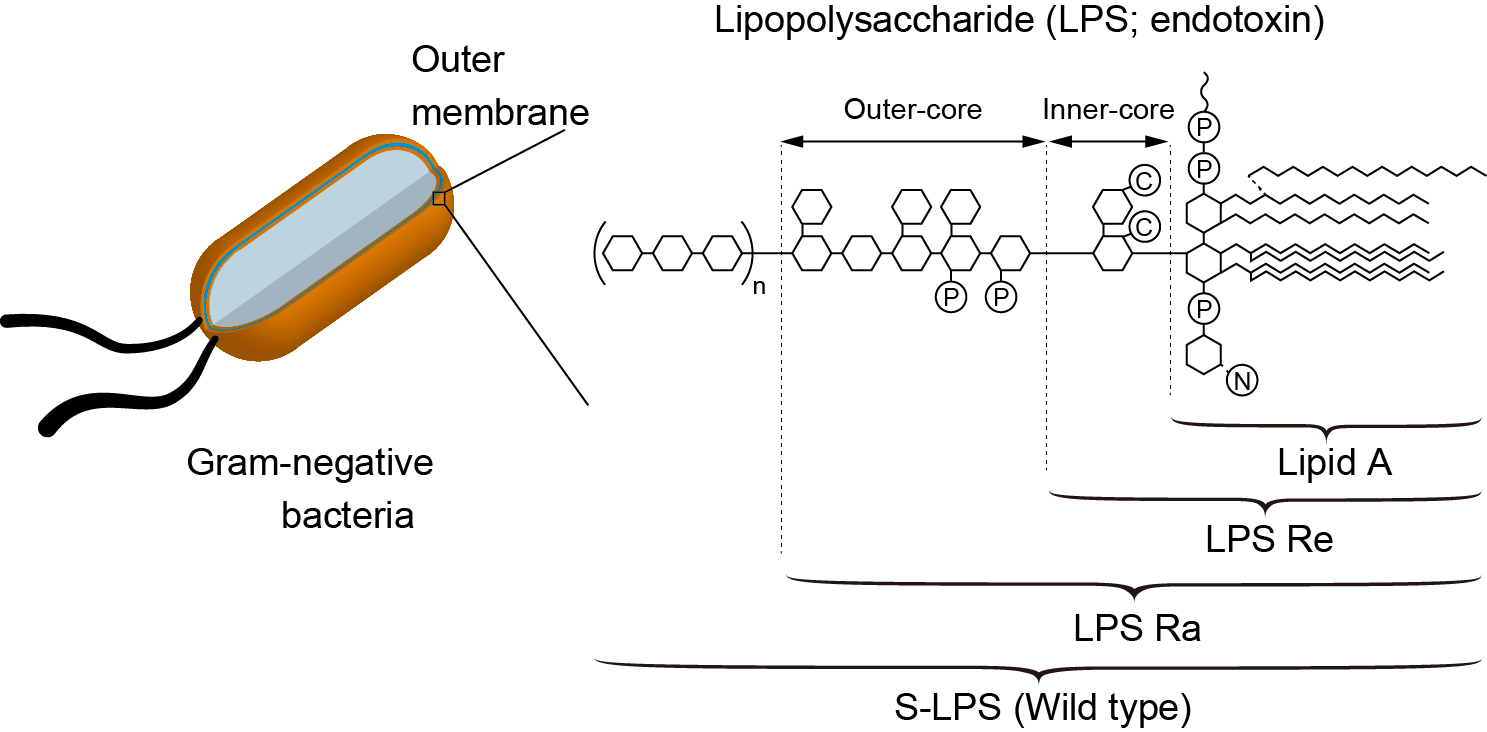


**Figure S4 | Schematics of lipopolysaccharides (LPSs, or endotoxin) molecules, which are contained in the outer membrane of Gram-negative bacteria (not to scale).** LPSs are called S-LPS (wild type), LPS Ra, LPS Re, and lipid A, depending on the length of saccharide chain in the hydrophilic group. LPSs cause septic shock, systematic inflammatory response syndromes, by altering the mechanics of erythrocytes.

**
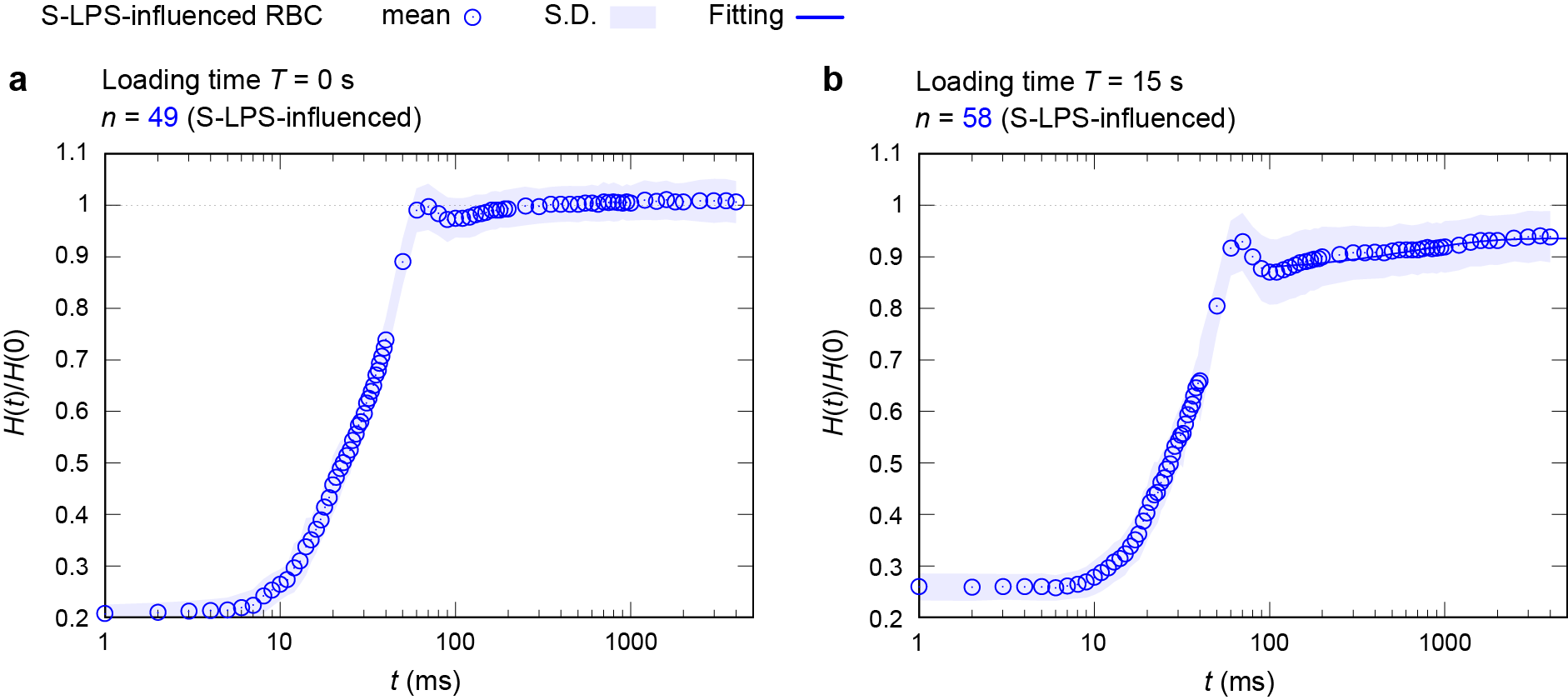
**

**Figure S5 |** Normalized cell heights for the loading times (a) *T* = 0 s and (b) *T* = 60 s as a function of recorded time *t* in (III) launching phase after the co-incubation with 100 μg/ml S-LPS. Mean values of the measured cell heights *H*(*t*)/*H*(*t*=0), the standard deviations, and the fittings with a theoretical function (only for *T* = 15 s) are represented by circular symbols, shades, and solid lines, respectively. Each number of samples *n* is given above the graph.


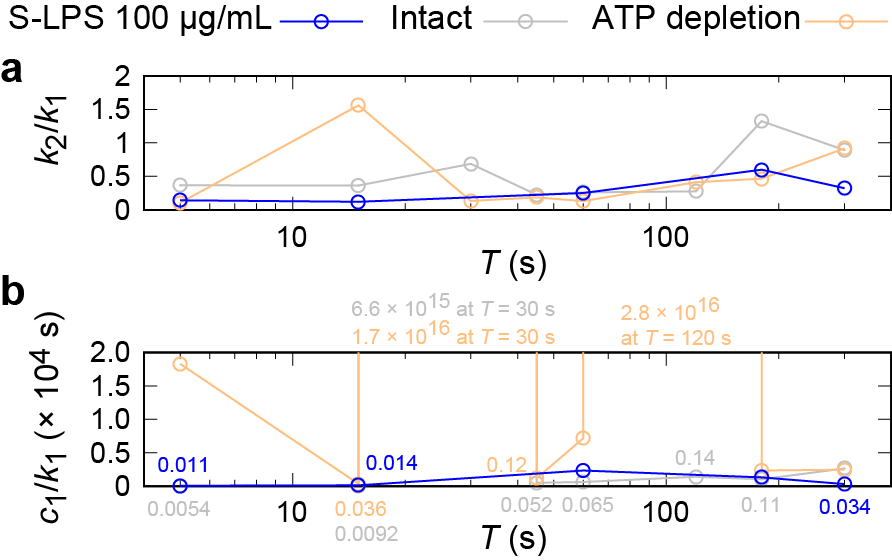


**Figure S6 | Estimated (a) *k*_2_/*k*_1_ and (b) *c*_1_/*k*_1_** of endotoxin-affected erythrocytes (blue) as a function of a widely ranged loading time *T* from second to hundreds of seconds. Results from intact (grey) and ATP depleted (orange) erythrocytes are underlain for comparison. *k*_2_/*k*_1_ and *c*_1_/*k*_1_ do not exhibit any clear systematic tendency among different loading time *T* and different metabolic conditions, contrary to *c*_2_/*k*_1_ and *τ* accompanied by the drastic transition by 2 orders of magnitude (see Fig.7).
